# Trauma and “spiritual resilience” among Internally Displaced Women (IDW) victims of violence in the commune of Dédougou, Burkina Faso

**DOI:** 10.12688/openresafrica.16517.1

**Published:** 2026-06-29

**Authors:** Yacouba Tengueri

**Affiliations:** 1 Sociology and anthropology, Daniel Ouezzzin Coulibaly University, Dédougou, Burkina Faso, BP 176 Dédougou, Burkina Faso

**Keywords:** Internally displaced women, violence, trauma, psychological care, Burkina Faso, spiritual resilience

## Abstract

**Context:**

Since 2015, Burkina Faso has been ravaged by the violence of armed terrorist groups (JNIM, EIGS), resulting in over 2 million internally displaced persons. In the Boucle du Mouhoun region, 63.17% of internally displaced persons (IDPs) are women (84,649 out of 133,994 in 2023). As prime targets of armed conflict, they suffer multifaceted violence used as a weapon of psychological warfare against communities.

**Methods:**

The study uses a convergent mixed-methods design conducted in October 2023 in the commune of Dédougou. The total sample size is 1,056 participants, including 1,015 IDPs surveyed using a digital questionnaire (KoboToolbox) and 41 individuals interviewed through semi-structured interviews. Quantitative data were analyzed using IBM SPSS 20, and qualitative data through thematic analysis.

**Results:**

The profile of the IDF reveals a majority of female farmers (51.4%), married (74.8%) or widowed (15.2%), with 43.9% having 4 to 6 dependent children. Statistically significant correlations were established between the year of displacement and physical (r = 0.074; p = 0.019) and psychological (r = 0.072; p = 0.022) violence. Nearly 46.3% of the IDF lost a loved one in horrific circumstances (throat-slitting, immolation, mass executions), resulting in severe post-traumatic stress disorder: chronic insomnia, flashbacks, mutism, and suicidal ideation. Faced with this trauma, prayer (39.74%) and silence (23.36%) constituted the main coping strategies. Despite the free psychological support provided by NGOs, the vast majority of IDFs do not access it, hampered by a lack of awareness of the services, their geographical dispersion, and a negative perception of psychological care.

**Conclusion:**

These results call for culturally grounded psychosocial interventions that integrate the spiritual dimension into therapeutic protocols and prioritize isolated widows and women with significant family responsibilities.

## Introduction

For the past decade, Burkina Faso has faced extreme violence from terrorist armed groups affiliated with Al-Qaeda (Jama’at Nasr al-Islam wal Muslimin, JNIM) and the Islamic State (Islamic State in the Greater Sahara, ISGS). The escalation of violence and the expansion of this crisis across 13 regions has generated approximately 2,062,533 internally displaced persons (IDPs), composed primarily of women and children. From 2020 to 2021, more than 1,425 violent events were recorded in 11 regions of Burkina Faso, with 51% of these attacks targeting the civilian population. In 2023, authorities counted approximately 133,994 IDPs (84,649 women and 49,345 men), representing 63.17% women in the Boucle du Mouhoun region ( MAHSN, 2025).

The magnitude of violence against the civilian population does not affect men and women to the same degree. Women are prime targets during crises because they are perceived as the future of society ( Santoire, 2020) or as social regulators through their positions as mothers, spouses, homemakers, and producers ( DCAF, OSCE/ODIHR, & UN Women, 2019; Foutem, 2021). Moreover, they are used as weapons or methods of psychological warfare against adversaries to leave an indelible mark ( Guignard, 2020). In Haiti, for example, Longtin (2010) describes the rape of opposition women by armed forces as a strategy of deterrence and family exclusion from political activities. From this perspective, Taxil and Massias (2024) analyze sexual violence during conflicts as a political construction whose primary objective is to symbolically humiliate men by desecrating the bodies of their women. Brownmiller (1975) characterizes this approach of systematic rape of women as a “conscious crime of intimidation” designed to render docile and terrorize men who cooperate with or join the regular army. However, Wood (2015) distinguishes between strategic violence and opportunistic violence. Strategic violence generally refers to execution of command hierarchy orders as a tactic of collective terror. Opportunistic violence, meanwhile, is not ordered by the hierarchy but represents personal initiatives by combatants.

Cohen and Nordås’s (2014) work on data from 1989 to 2009 from the Sexual Violence in Armed Conflict platform found that 32% of 117 armed actors systematically resort to sexual violence. For these authors, this widespread sexual violence is explained by the weakness and fragmentation of armed groups’ command chains, forced recruitment of combatants, absence of internal justice mechanisms in these armed groups, and total impunity for perpetrators of sexual violence. This violence and absence of justice mechanisms should not be analyzed as a loss of control by the command hierarchy over combatants but rather as payment in kind and a pledge of cohesion between the hierarchy and combatants.

In the Nigerian context, school-age girls are the primary targets ( Zenn, 2018) of the Boko Haram terrorist armed group. Indeed, the Federal Ministry of Education (2021) highlights in its work the kidnapping of 200 girls from Chibok secondary school in 2014 and 100 other schoolgirls in 2018 in the village of Dapchi. These girls are sometimes used as human shields during regular army assaults, as kamikazes ( Barna, 2014; Bloom & Matfess, 2016), or as “human bombs” ( UNODC, 2017). These kidnappings are sometimes analyzed as retaliatory measures against the detention of family members of Boko Haram armed group combatants by the Nigerian state. However, research shows that these hostage women are sometimes used as intelligence and surveillance agents for new hostages to brainwash them ( Bloom & Matfess, 2016) or as cooks and weapons carriers ( UNODC, 2017). These women are integrated into their apparatus to capitalize on the element of surprise against regular forces. Beyond this, the sexual slavery to which they are subjected under the banner of forced marriage fits within the perverse logic of creating a new generation of combatants who would benefit from socialization anchored in the group’s Islamist ideology ( Bloom & Matfess, 2016).

When they escape from armed groups, they are stigmatized by the community because children born from these non-consensual relations are labeled as “bad blood” ( UNODC, 2017). This social disqualification renders these women, already traumatized by years of captivity, even more vulnerable, thereby facilitating their return to these armed groups. Indeed, according to Peterman et al.’s (2011) study, more than 1.69 million women in the Democratic Republic of Congo (DRC) were rape victims. These Congolese women suffered genital mutilations, public humiliations, and carried rape pregnancies ( Eriksson Baaz & Stern, 2013). Unlike Congo, in Iraq, Daesh subjected women to sexual slavery, sold them, or gave them as gifts to combatants. These figures appear to underestimate reality according to work by Hynes et al. (2004) and De Oliveira Araujo et al. (2019) because the subject remains sensitive and touches victims’ intimacy. Consequently, very few women report for fear of being rejected or stigmatized.

Burkina Faso, Mali, and Niger face the same reality experienced by women in Nigeria, DRC, and Iraq. Indeed, according to the CONASUR (2023) report, women are victims of sexual violence and psychosocial distress. While the literature is abundant on sexual violence during conflicts, this article aims to determine the different forms of violence, their consequences on internally displaced women’s (IDW) mental health, their psychological care, and resilience strategies developed by IDW.

## 1. Methodological approach

### 1.1. Study coverage area

The city of Dédougou is a department and an urban commune located in the Mouhoun province of Burkina Faso. According to the RGPH (2019), its population is estimated at 123,934 inhabitants. The commune of Dédougou shares borders with the following communes: to the east with the commune of Douroula, to the west with Sanaba and Bourasso, to the north with the communes of Sono and Gassan, and to the southeast with the commune of Safané. Administratively, Dédougou is subdivided into seven sectors and 37 villages. It brings together several ethnic groups (Bwaba, Marka, Fulani, Mossi, Samo, etc.), with the main languages spoken being Bwamu and Jula. Its economic activity is based primarily on agriculture and livestock farming. Map 1 indicates the area covered during data collection.

**Map 1.**
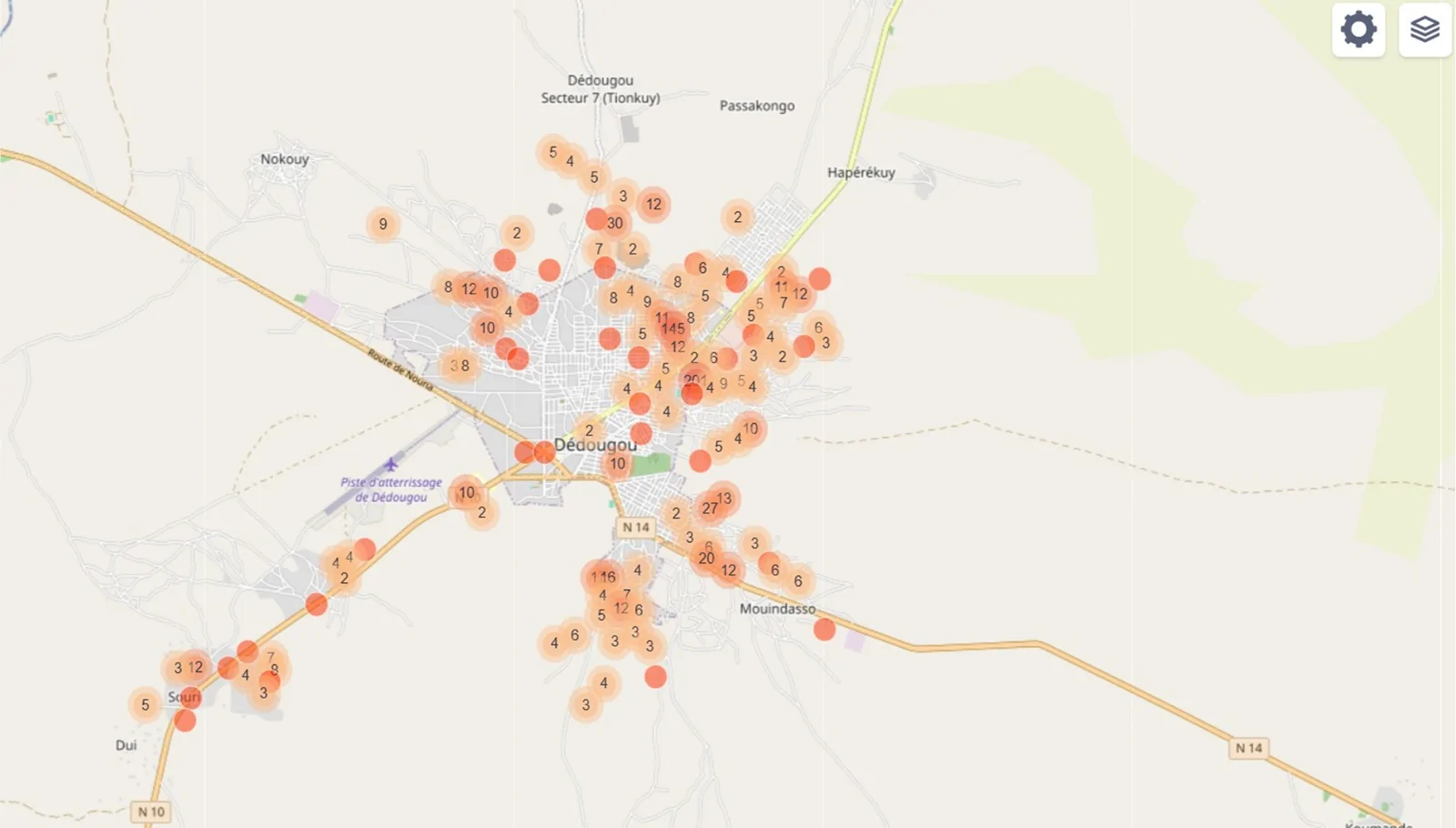
Area covered by the survey.

### 1.2. Study population

The surveyed population consisted primarily of all internally displaced women (IDW) located on official and unofficial sites. Women victims of terrorism constitute the target population of our study in the city of Dédougou. Additionally, spouses, adult children, and close relatives constituted the control population. The survey was also extended to resource persons including non-governmental organizations (NGOs) operating in the area and state structures (Social Action).

### 1.3. Methods

The study of psychological and social care for women victims of terrorism is a complex object. According to Creswell and Plano Clark (2017), understanding and explaining complex social phenomena requires an integrative approach. Therefore, to grasp the scope and complexity of this theme, we opted for a convergent mixed design ( Fetters et al., 2013). This involved independent and simultaneous collection of quantitative and qualitative data. The quantitative data aimed to capture the scope and nature of the violence suffered as well as its traumatic manifestations. The qualitative approach aimed to capture the lived history of violence and the therapeutic pathway of violence victims.

### 1.4. Data collection tools and techniques

To work on a subject as sensitive as violence against IDW, we constructed two categories of tools. The questionnaire was developed using KoboToolbox, a digital tool adapted to field surveys, facilitating data entry, centralization, and quality control of collected information. The questionnaire was structured into three main parts: information about the respondent (village of origin, year of departure, number of children, marital status, and profession before departure), types of violence suffered and types of trauma felt, and preferred therapeutic pathway.

Semi-structured interviews conducted with women victims of terrorism were carried out using an interview guide. This technique aimed to collect information on the causes of displacement, identify multiple forms of violence suffered (physical, psychological, verbal, economic, and sexual) before and during flight, the lived experience, and therapeutic pathway for this violence.

### 1.5. Sampling and sample

In this quantitative study, we used two sampling techniques due to the geographic dispersion (see Map 1) of the majority of internally displaced persons: a combination of simple random sampling and snowball sampling. The absence of a sampling frame or exhaustive list of internally displaced persons led to privileging this mixed approach to constitute the quantitative sample. Indeed, the simple random sampling technique on official sites ensured statistical representativeness. The snowball sampling technique borrowed from the qualitative method identified those who had resettled in informal habitats or were housed with guardians. This strategy aimed to guarantee complete and representative coverage of IDPs in the Dédougou commune given their geographic dispersion. As for qualitative sampling, purposive sampling was privileged with inclusion criteria based on categories concerned by the interviews. The main inclusion criterion was internally displaced status for women, for NGOs working in the humanitarian field, for state agents being from the Ministry of Social Action, and for mental health professionals working in the humanitarian sector. Triangulation and information saturation led to ending our interviews after observing constancy. Table 1 summarizes the quantitative and qualitative sample.

**Table 1.** Quantitative and qualitative sample.

Survey type	Sampling type	Survey population	Sample
Quantitative survey	Simple random	IDW	**1015**
Qualitative survey	Purposive	Spouses	**25**
		Women victims of violence	**11**
		NGOs (ASMADE, INTERSOS, OCADES, Médecins Sans Frontières)	**04**
		Social Action	**01**
TOTAL			**1056**

*Source:* Field survey, October 2023.

### 1.6. Analysis methods and informed consent

The qualitative data gathered from our empirical investigations were manually transcribed and analyzed through thematic content analysis, ensuring fidelity to respondents’ statements. We conducted thematic content analysis of verbatim responses following the method of Braun and Clarke (2006). The use of respondents’ statements for analytical purposes adhered to ethical principles and professional standards in the social and human sciences in the absence of an institutional ethics committee. We took care not to use participants’ real identities. Additionally, before beginning each interview, participants’ free and informed consent was obtained. Furthermore, the sensitive nature of the subject required isolation of participants to access their intimate experiences and guarantee their physical and moral safety. The quantitative data collected via the KoboToolbox platform were exported to IBM SPSS Statistics 20 software for variable cross-tabulations and Pearson correlation calculations. Microsoft Excel 2020 was used to generate statistical tables.

Moreover, this study draws on Cyrulnik’s (2001) resilience theory and Collins’s (2019) intersectionality theory as an analytical framework for gender inequalities in adaptive capacity and the differentiated impact of trauma related to violence perpetrated by terrorist armed groups. IDW, not possessing the same social capital, develop differentiated adaptive strategies to context ( Rutter, 1987) to heal their emotional and physical wounds. For Cyrulnik (2001), the role of social and affective bonds and the individual’s inner liberating strength play a crucial role in individual reconstruction.

### 1.7. Ethics statement

This study was conducted in accordance with the ethical guidelines commonly applied in social science research. Prior to the start of each interview, the free and informed consent of all participants was obtained. However, most of the participants involved in this research are largely illiterate, and in the local context signing a document is often perceived as a form of binding commitment. This perception can generate fear or reluctance among participants. For this reason, verbal consent was considered the most appropriate and ethically respectful procedure. Participants were clearly informed about the objectives of the study, the voluntary nature of their participation, and their right to withdraw at any time. It is also important to note the specific social context of the study. Many participants depend largely on assistance from the state and non-governmental organizations, and external actors are often perceived as potential sources of support. Under such circumstances, there is a risk that research activities may be misinterpreted as linked to aid distribution. To avoid creating unrealistic expectations or misunderstandings, particular care was taken to clarify the purpose of the research. Despite these precautions, this context also partly explains the initial reluctance of some individuals to participate in the survey.

## 2. Results

This section presents the profiles of women victims of violence, the correlations between displacement and violence intensity, the lived experience of violence by IDW, and finally their resilience capacity facing the trauma suffered.

### 2.1. Profile of Internally Displaced Women (IDW)

The analysis of individual variables such as marital status, profession, and number of dependent children aims primarily to capture their vulnerability to violence. Indeed, we start from the postulate that widows, youth, the unemployed, and those with numerous dependent children are vulnerable social categories. Indeed, Table 2 below indicates that the vast majority of IDW were farmers (51.4%), 22.4% homemakers, 20% traders. A minority had occupations such as sewing, gardening, hairdressing, craftwork (weaving, pottery, etc.), community health aide, etc. A small proportion of women were involved in livestock raising, only 0.3%. However, after forced displacement, the vast majority of displaced persons struggle to exercise their former profession in the new refuge zone. Sociocultural constraints, land scarcity, and monetization of land transactions in the Dédougou commune impede their access to agricultural land portions to practice subsistence agriculture. Consequently, these IDW reconvert for the most part to collecting gravel, sand, dead wood, plastic bags, and begging to meet family members’ needs.

**Table 2.** Professional status of IDW.

Professional status	Frequency	Percentage
Farmer	522	**51.4**
Homemaker	227	22.4
Trader	203	20.0
Student	22	2.2
Seamstress	12	1.2
Gardener	7	0.7
Hairdresser	6	0.6
Artisan	5	0.5
Community health aide	4	0.4
University student	4	0.4
Total	**1,015**	**100.0**

*Source:* Field survey, October 2023.

This Table 3 indicates that women with married and widow status constitute the most important category. They have 90% of the children under their care. Married women represent 74.8% of the sample and widows 15.2%. The other categories have 10% of children under their care. However, 43.9% of IDW have between 4 to 6 children under their care, 27.9% between 1 and 3 children, 16.6% have between 7 to 9 children. Those with more than 10 children under their care represent 3.7% of the sample. Moreover, it emerges that 1.95% of widows have no children under their care. This category of widows is more vulnerable emotionally as they have no attachment at the family unit level. They are more exposed to trauma effects if they do not receive adequate care. A significant number of these women became widows following attacks.

**Table 3.** Number of dependent children by marital status of IDW.

Number of dependent children	None	1–3	4–6	7–9	10–12	13–19	Total	Freq	P%
Single	43	18	1	2	0	0	64	64	6.3
Cohabiting	2	15	7	2	1	1	28	28	2.8
Divorced	0	3	4	2	1	0	10	10	1.0
Married	32	224	355	122	23	3	759	759	**74.8**
Widow	**3**	23	79	40	9	0	154	154	15.2
Total	80	283	**446**	168	34	4	1,015	1,015	100

*Source:* Field survey, October 2023.

### 2.2. Escalation and temporal intensification of violence

The IDW surveyed on official sites and in unarranged peripheral neighborhoods originate from about one hundred villages in the Sourou and Bankuy regions. However, the vast majority come from Djédougou or Guédougou, Gassan, Sanaba, and Douroula and underwent forced displacement from 2017 to 2023. The peak of forced displacement occurred in 2022 followed by a decline in 2023. All IDW, 100%, justify their displacement by violence and ultimatums from terrorist armed groups. Communities received short deadlines of 24 hours or 3 days to evacuate the village at risk of execution for disobedience.

Table 4, represented in Figure 1, determines the Pearson relationship between certain temporal variables and types of violence. Here, correlation is significant with two variable types (physical and psychological). The sample size ( *N*) for the year of displacement variable is 1,015 IDW. It emerges from Figure 1 that the correlation is statistically significant between physical and psychological violence. Indeed, there is a significant positive correlation ( *r* = 0.0738, *p* = 0.0186) between year of displacement and physical violence. These data reveal that the more recent the displacement, the greater the probability and intensity of physical violence increases, or inversely. This correlation is significant between year of displacement of IDW and psychological violence ( *r* = 0.072, *p* = 0.0219). However, there is a significant negative correlation ( *r* = −0.0498, *p* = 0.1126) between year of displacement and social violence (exclusion, rejection). This significance suggests that the more recent the year of displacement, the less they feel rejected or socially excluded, or inversely. These data allow us to affirm that over the years, IDW are perceived positively by the host population.

**Table 4.** Pearson correlations between year of displacement and types of violence.

Type of violence	Correlation coefficient ( *r*)	*p*-value	Interpretation
Physical	0.0738	0.0186	Significant correlation (p < 0.05)
Psychological	0.072	0.0219	Significant correlation (p < 0.05)
Sexual	−0.0172	0.5834	No significant correlation
Economic	0.0103	0.7432	No significant correlation
Verbal	0.0198	0.5285	No significant correlation
Social (exclusion, rejection)	−0.0498	0.1126	No significant correlation

*Source:* Field survey, October 2023; IBM SPSS 20.

**Figure 1.**
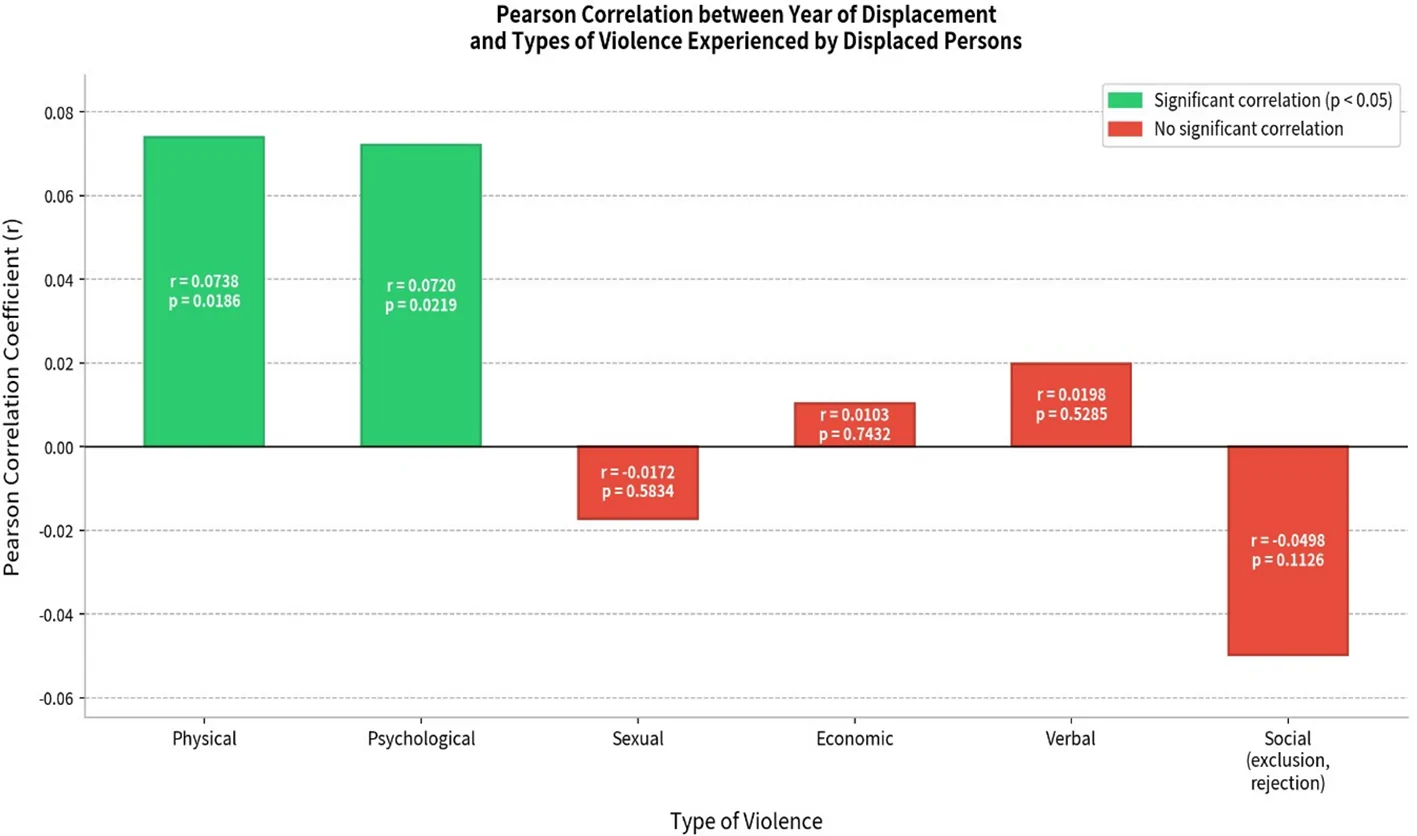
Pearson correlation.

In addition to these types of violence, Table 4 allows us to affirm that correlations between year of displacement and sexual violence ( *r* = −0.0172, *p* = 0.5834), verbal ( *r* = 0.0198, *p* = 0.5285), and economic ( *r* = 0.0103, *p* = 0.7432) are not statistically significant because *p*-values are greater than 0.05.

Table 5 above reveals that there is a moderate significant positive correlation between physical violence and psychological violence ( *r* = 0.5006). Those who are victims of physical violence tend to suffer verbal violence ( *r* = 0.4297). Persons suffering from physical violence are also victims of social exclusion.

**Table 5.** Pearson correlations among types of violence.

Type of violence	Physical	Psychological	Sexual	Economic	Verbal	Social
Physical	1	0.5006	0.1915	0.2331	0.4297	0.2113
Psychological	0.5006	1	−0.0075	0.1968	0.286	0.2818
Sexual	0.1915	−0.0075	1	−0.03	0.1724	−0.0238
Economic	0.2331	0.1968	−0.03	1	0.1098	0.2188
Verbal	0.4297	0.286	0.1724	0.1098	1	−0.0118
Social	0.2113	0.2818	−0.0238	0.2188	−0.0118	1

*Source:* Field survey, 2023.

### 2.3. Sophisticated terror strategy: The lived experience of violence by women

These studies examine violence experienced by IDW before, during, and after displacement. Violence suffered by most IDW before forced displacement included deprivation of liberty and imposition of strict laws to govern behaviors according to Islamic precepts. Under the control of terrorist armed groups in their villages of origin, inhabitants were under threat of execution, beatings, prohibitions against playing music, consuming millet beer, playing soccer, etc. Physical violence included imposition of the hijab or black robes to cover their entire bodies. Those who did not respect these dress codes were beaten, humiliated, raped before their spouses.

However, most atrocities were committed during attacks and forced displacement. Table 6 indicates that 46.3% of IDW lost at least one family member (spouse, children, brothers, sisters, brother-in-law, father-in-law) or community member during attacks. Men were shot in the head, others had throats slit, others were thrown into wells or burned alive. Sometimes, men were gathered in a single location to be executed en masse before women and children, as was the case with residents of the village of Guédougou. In this context of extreme violence, some families were decimated. Women’s narratives were marked by silence, tears, bitter laughter, interjections. Men, regardless of age, were systematically targeted for killing. This “scorched earth” policy strategy aimed to deter any collaboration or enlistment of men in the Volunteers for the Defense of the Homeland (VDP) auxiliary force. Moreover, forcing women and children to witness the murder of their loved ones fit within an electro-shock and deterrence policy. This is the case of Respondent 17 who finds herself the sole survivor of her family in the city of Dédougou:

**Table 6.** Human losses before and during forced displacement.

Did you lose a family member during the attack	Frequency	Percentage
No	545	53.7
Yes	470	46.3
Total	**1,015**	**100.0**

*Source:* Field survey, October 2023.

Me, in any case, I no longer know if I am human. I live today just to live. If you kill yourself, they will accuse you of having committed a crime. Otherwise, I prefer to be dead than alive to have peace. When our village was attacked, I lost twelve members of my family the same day. I lost my husband, my children, and my husband’s brother. The terrorists’ targets were boys. They came one morning and killed most of the boys. When they attacked the village, my husband had already left with our son to go fishing in the river. They were killed. I did not even see their bodies. Currently, I live alone here.

In addition to this victim, Respondent 21 was instead forced with her children to witness her husband’s execution. Indeed, she recounts the lived experience of this day of atrocity in these terms:

I am from Koumbara. We were stopped and stripped by the terrorists during our flight from our village. Then, they slit my husband’s throat before my eyes and my children. They forced us to walk to reach where we wished to take refuge. At one point, they wanted to burn us alive. I was lost, desperate. I took the road on foot with my three children to Dédougou here.

Respondent 4 experienced similar violence. Indeed, she was instead forced to witness the slitting of her only son’s throat. Through her narrative, it emerges that victims also suffer moral torture and insults. Verbal constraint is exercised on them not to manifest their mourning and express their pain. It emerges from her words, the atrocity of violence in these terms:

I come from the village of Bao. My eldest son had his throat slit before my eyes because he refused to join them. He was killed on a Wednesday. I went mad, I cannot overcome this cruelty. I cried so loud that “those people” (referring to terrorists) came back to tell me to shut my mouth or they would burn me alive. I responded that they could kill me. They came back the following Friday, giving us an ultimatum to leave the village in the afternoon. They formally forbade us from going to Tougan which was the nearest village otherwise we all risked dying. I had lost reason with the loss of my only son who took care of me and his six sisters since their father’s death *.*

Unlike these violence victims, Respondent 4 from the village of Bouna was forced with her husband into infanticide and cannibalism. The extreme trauma of this day of violence is recounted by the victim in these words:

I come from Bouna. During our displacement here, we did not suffer violence. It was rather before we displaced… About three years ago, one afternoon, armed individuals came to our village. They told inhabitants to leave immediately. I was almost at term. My husband told me we could not keep pace with the others… We stayed and at 7 p.m. we started walking, a few steps from the house I started losing water. We returned to the house to give birth. We rested a bit and the next morning very early, when we were preparing to leave, they landed at our house, they asked us why we did not leave, that we did not receive the order to leave the village, no? My husband responded that we received it, but that we could not leave because I was at term and I just gave birth. They said nothing, but they went out to confer for a good while and one of them came back ordering my husband to take the baby, slit its throat, cut it into pieces, and cook it. Hmm, we were so shocked, devastated, it is inhuman. We begged them in vain. Oh Lord! I cried, my husband even was overwhelmed by the situation. He lost the power of speech, he could not even speak. The worst is that after cooking, they wanted to force us to eat the meat and soup of our baby (cries). It was like a bad dream yet it was real. In fear of being next, we started serving the soup (our baby’s flesh), the Defense and Security Forces arrived in time to prevent them from committing this ignominy. There were exchanges of gunfire, thank God our FDS prevailed and we got out unharmed (cries).

An analysis of this verbatim reveals trauma among women exposed to violence experienced during assassinations of their husbands and children. The execution of spouses, children, and acquaintances left deep mental scars in certain IDW. This trauma manifests through mental disorders, loss of speech, insomnia, repetitive nightmares, anxiety, flashbacks, and tears. Some end up feeling “crazy” or desperate to the point of considering suicide as an alternative to eradicating mental suffering.

Establishing family relationships with IDW highlights that certain categories are more affected than others. Indeed, Table 7 indicates that the category of spouses (17.45%) and children (19.96%) constitutes 37.41% of human losses. This category represents an essential part of the family structure and the one with which IDW have a direct link. Brothers/Sisters constitute 25.90%, a quarter of human losses recorded during attacks and kidnappings. The parents category represents 21.94% and 14.75% of neighbors and acquaintances.

**Table 7.** Type of relationship with victims.

Relationship with victim	Frequency	Percentage
Spouse	97	17.45
Child	111	19.96
Parent	122	21.94
Brother/sister	144	25.90
Others	82	14.75
Total	**556**	**100.00**

*Source:* Field survey, October 2023.

### 2.4. Spiritual resilience and post-traumatic psychological care

Facing violence, IDW adopt several attitudes or self-protection mechanisms. These protection mechanisms vary from one actor to another. Figure 2 indicates that 39.74% of women take refuge in prayer sessions as a therapeutic strategy. They depend largely on this type of spiritual support mechanism to manage stress and trauma related to extreme violence experienced. Faith and prayer function as a refuge and allow many victims to find inner peace and give meaning to tragic events. Women console themselves by placing their fate in God’s hands. Non-religious strategies such as silence (23.36%) and support from loved ones (17.48%) appear as protection mechanisms against violence. Silence indicates internalization of trauma, difficulty externalizing suffering, or the conviction that opening up to expose one’s resentments appears dangerous. Unlike this silence, those who confide in loved ones indicate the importance of immediate social capital in the healing process of inner wounds.

**Figure 2.**
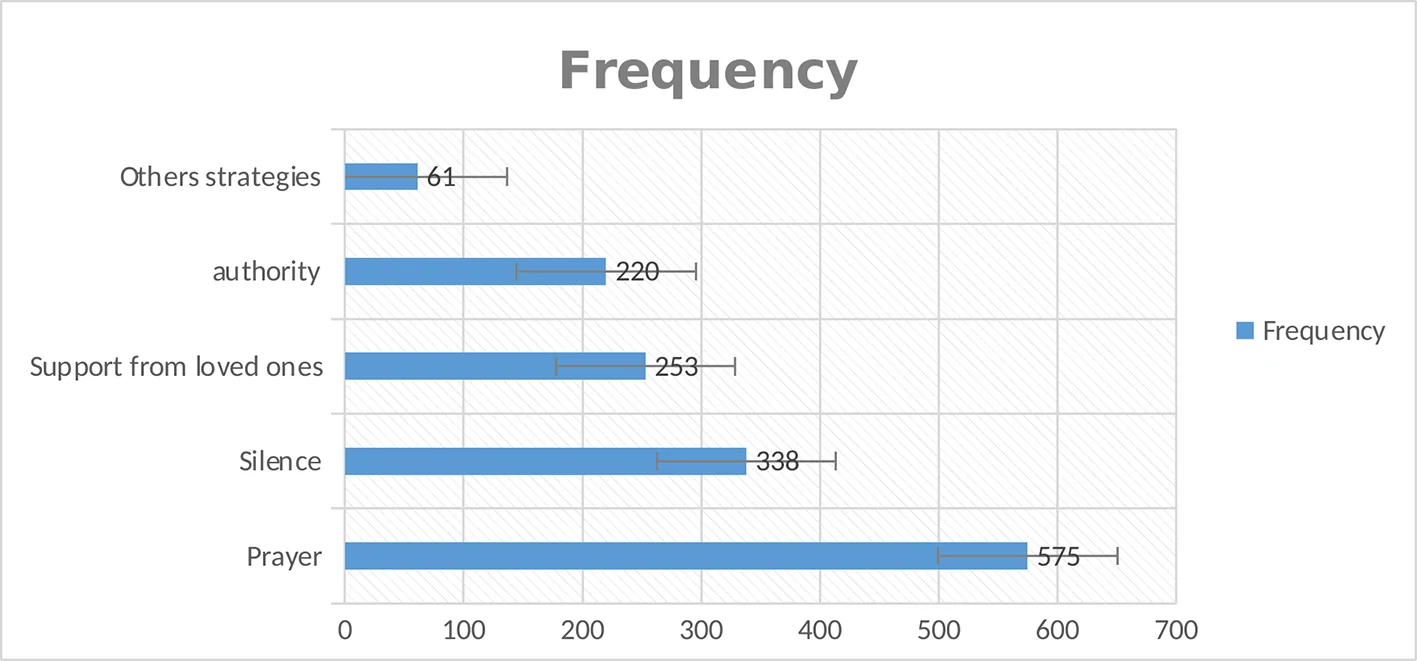
Recourse facing violence.

A small percentage of women resort to authorities, only 15.2%. This situation is due to unfamiliarity with institutions empowered to emotionally support victims of psychological violence. Only 4.22% resort to strategies such as throwing themselves into work and recourse to traditional remedies. Indeed, collecting gravel and dead wood in forests appears as therapy for women. The occupation allows drowning negative thoughts (suicidal thoughts) and overcoming the pain of tragic events experienced. Another strategy used by women victims of violence is the use of decoctions (herbal teas, bark, etc.) to rid themselves of lost souls of their loved ones who haunt them. Facing mental trauma and illnesses, these strategies appear as an alternative to modern medicine which requires more financial means.

Indeed, from semi-structured interviews, it emerges that IDW rarely resort to psychologists for accompaniment in healing psychological wounds. However, certain IDW installed on official sites benefit from accompaniment and follow-up by psychologists. Awareness sessions are organized by social action agents, nurses, and NGOs (Red Cross, Médecins Sans Frontières, or INTERSOS). Care consists of conducting home visits to victims by performing psychological exercises, educational awareness sessions, discussions, spiritual and emotional support. This appears in the words of Respondent 7 (Psychologist at an NGO) in these terms:

Persons who are victims of terrorism need awareness, psycho-education, individual interviews, relaxation therapy. With these different aids, the individual manages to overcome their disorders and also adapt to a new way of life *.*

This also emerges from the words of Respondent 32: “Once, NGO agents came to talk with us to encourage us. This helps us forget our fears and our traumas.” Respondents recognize free care for psychological health problems. However, a vast majority of IDW affirm not resorting to or consulting psychologists made available to them in housing camps. Contrary to this information, NGOs like Médecins Sans Frontières affirm receiving around 30 to 40 persons per week with post-traumatic disorders for complete care. This free care is mentioned by Respondent 3 (agent of a humanitarian NGO) in these words:

Care for persons who are victims of terrorism is free. After receiving them, we make a diagnosis to know the state and intensity of the harm they suffer. We also make a follow-up program for the person and a care plan for the sick person *.*

A comparative analysis of verbatim from humanitarian workers and women victims of trauma reveals that IDW are unaware of the existence of free psychological care. Indeed, they announce financial difficulties as a barrier to consulting a psychologist. For other categories, their priority is ensuring children have food so as not to die of hunger. The latter are convinced that these psychological consultations will not bring back loved ones lost in attacks.

## 3. Discussion

Our empirical investigations reveal a reality observable in all conflict-infested zones, whether symmetric or asymmetric: women become a terrain of political and media confrontation. In certain conflicts like Vietnam, the slogan was “Occupy women’s wombs” ( Maquestiau & In’t Zandt, 2017). Indeed, in conflict zones, they are more exposed to risks of pregnancy, sexually transmitted diseases, and social disqualification as highlighted in work by Longtin (2010), UNODC (2017), and Taxil and Massias (2024). Very few IDW admit having suffered different forms of sexual violence because it is a risk of being ineligible for marriage or being socially excluded ( Hynes et al., 2004; Bouvier, 2014; Maquestiau & In’t Zandt, 2017; De Oliveira Araujo et al., 2019; Tengueri, 2026a). The sociocultural context and negative perception of rape explain this negative correlation ( *r* = −0.0172; *p*-value = 0.5834). Most often, they wall themselves in silence and choose to die with the secret of rape so as not to appear as beings soiled by the enemy ( Bouvier, 2014) or be rejected by family and community. Moreover, this study indicates a significant positive correlation between physical, psychological ( *r* = 0.5006), and verbal ( *r* = 0.4297) violence. This indicates that victims suffered physical, verbal, and psychological violence. Indeed, during attacks, women were forced to wear the hijab, were beaten, insulted, and humiliated in public.

The absence of significant correlation regarding social violence ( *p* = 0.1126) corroborates results obtained by OCADES (2022) within the framework of the PARJEF-PDI project in the North and Boucle du Mouhoun regions. This report also indicates that the majority of households appreciate their relationship with the host population. Indeed, 56% estimate that there is cohesion between host households and IDPs. However, for FAO (2021), host population vulnerability and other factors constitute major risks that can lead to conflicts and deterioration of social cohesion with displaced persons. Consequently, it proposes addressing grievances, inequalities, marginalization, violence, and existing interethnic tensions before resettling forcibly displaced persons.

Trauma symptoms due to exposure to extreme violence in Burkina Faso are similar to observation data from Cetorelli et al. (2017) among Yazidi women in Iraq. In both contexts, women witnessed execution of their spouses and male children. Consequently, they become insomniacs, develop suicidal feelings, lose power of speech, and develop feelings of madness ( Elbert et al., 2013). Moreover, we also note neurovegetative hyperactivation (hypervigilance and chronic insomnia), traumatic re-experiencing (“the slitting of my son’s throat haunts me,” “I see my son burned alive again”).

In terms of therapeutic pathway, our results reveal that women resorted to prayer (39.74%) and silence (23.6%) as post-traumatic disorder management strategies. These results are similar to findings by Ouedraogo (2019) on the therapeutic pathway of persons with mental disorders in the North region of Burkina Faso. From this perspective, Panter-Brick and Leckman (2013) suggest integrating a culturally anchored resilience approach to care. Integrating prayer into psychological disorder care would strongly contribute to improving victims’ mental health status. However, systematic disqualification of this prayer-based approach in favor of standardized interventions does not provide a culturally anchored response. Taking this reality into account by NGOs and the Ministry of Social Action in their diagnostic and care approach would contribute to significantly improving women’s condition. Moreover, our study reveals that despite free psychological care, very few women resort to it. Free care and psychologist availability in displaced persons’ camps does not guarantee professional care attendance rates. This low recourse is explained primarily by four interacting factors: geographic dispersion of IDW, ignorance of psychological services’ existence, perception of these services as the “madhouse,” and inversion in need hierarchy compared to Maslow’s classification. They place their food needs before their psychological well-being. For others, consulting a psychologist is not culturally anchored in habitus and most often, users of these services are labeled “white” or fragile.

In the same vein, Summerfield (2001) explains that systematic imposition of the DSM approach in societies with another conception of person and suffering appears ineffective in terms of expected responses. The author emphasizes that in these non-Western societies with different nosologies from Western society, psychological distress is sometimes perceived as spiritual possession or a response to transgression of ancestral prohibitions, etc. Moreover, this is the conviction of some of our respondents who affirm having used decoctions for purification baths to rid themselves of haunting by suffering souls of loved ones killed in atrocious conditions. Modern medicine cannot provide a response to “red deaths,” an IDW told us. These beliefs escape biomedical Western reading grids. Studies by Betancourt et al. (2015, 2010) in Sierra Leone and Patel et al. (2018) in Zimbabwe demonstrate that child soldiers’ participation in deliverance prayers and community therapy groups have a positive effect on children’s anxio-depressive symptoms. However, our results show limits in “spiritual resilience” adopted by IDW confronted with cannibalism, infanticide, or extermination of all family members. The expression “I live just to live” reveals their dehumanization, their loss of taste for life. This category of victim having no more emotional attachment, if not assisted and followed, is most inclined to suicide ( De Jong et al., 2001; Steel et al., 2009) or “fatalistic suicide” ( Durkheim, 2005). Our results join conclusions of work by Elbert et al. (2013) on dehumanization and loss of existential meaning characteristic of Post-Traumatic Stress Disorder (PTSD).

However, adoption of silence by certain IDW as an individual defense mechanism to attenuate trauma appears risky in the long term. Indeed, in our context, 23.6% of IDW resort to this strategy. However, for Tol et al. (2013), this therapy approach allows short-term response to traumatic situation survival but constitutes a danger for the person long-term. For this author, it is a solution potentially carrying chronic long-term disorders. These persons present a risk of depression evaluated at 2.5 times compared to persons confiding in a PTSD professional ( De Oliveira Araujo et al., 2019). Follow-up of this vulnerable IDW category appears urgent as they are predisposed to relapse.

An intersectional analysis of sociodemographic profile reveals that IDW are not a homogeneous group. Their vulnerability varies according to marital status, professional status, and family burden. Indeed, widows representing 15.2% of the sample, 43.9% have 4 to 6 children under their care, and 51.4% were farmers. However, those who held small agricultural exploitations in their village of origin to meet some of their food, economic, clothing, and health needs struggle to access a land portion in the reception zone. Indeed, Tengueri’s (2023) study reveals that land scarcity, land transactions, sociocultural constraints, and perception of IDPs as privileged (food donations, cash transfers, free care in health facilities, etc.) complicate their right to access an agricultural land portion with the host population. In the trauma context, work appears as a refuge to escape depression. This inability to exercise former occupations in the reception zone renders their economic, social, and psychological reconstruction even more vulnerable. They are victims of double trauma: the mental burden of meeting children’s food and health needs but also the loss of spouses, children, and loved ones in inhuman conditions. Those who lost an immediate family unit member represent 37.41% of human losses. This reality reflects the collapse of family support structures in grief situations but also their socioeconomic reference point ( HRW, 2019). Indeed, our results indicate that 46.3% of IDW lost a loved one, which constitutes an alarming indicator of human cost. In the Democratic Republic of Congo, Peterman et al. (2011) also found that women lost approximately 40% of their immediate loved ones in violence. An intersectional analysis reveals differential vulnerability between widowhood status and motherhood. Indeed, according to Hynes et al. (2004), widows present 3.2 times greater risk of sinking into psychological distress than married women in the same situation. The 1.95% of widows who lost husband and child in the crisis are most often invisible as they have no family, economic, and emotional support network. This widow category is more vulnerable and runs greater risk of slipping into chronic depression. Facing this reintegration reality, IDW reconvert for the most part to collecting gravel, sand, dead wood, plastic bags, and begging to meet their family members’ needs.

## Conclusion

The study on resilience of women victims of terrorism in Burkina Faso highlights the multiform and systematic extreme violence to which they were exposed before and during forced displacement. They were victims of physical violence (mutilation, beatings), psychological violence (cannibalism, infanticide, exposure to execution of loved ones, public humiliation, etc.), and sexual violence (touching, rape). This deliberate brutality maintained by terrorist armed groups against the civilian population fits within a logic of spatial marking through mediatization of violence. Extreme violence serves to deter men, women, and youth from enlisting or collaborating with defense and security forces. Implementation of the “scorched earth” policy consisted of systematically executing men and male youth. This human tragedy has consequences on the social, economic, and vulnerability levels of IDW. Indeed, these victim women present signs of post-traumatic disorders. Most have chronic insomnia, flashbacks, nightmares, madness, loss of speech, isolation, tears, etc. Moreover, this research highlights vulnerability according to marital status and family burden. Indeed, childless widows, women with 4 to 6 years under their care, whether widows or not, present differentiated vulnerability risks. However, if they are cared for by psychologists from humanitarian NGOs and Social Action in internally displaced persons’ camps, many escape their coverage due to their geographic dispersion, ignorance, and negative perception of psychologists. The vast majority turn to prayer and silence to face trauma. These results therefore suggest contextual intervention anchored in cultural nosologies of post-traumatic disorders with priority targeting of isolated widows and women with numerous children under their care. Moreover, accompanying IDW in exercising income-generating activities appears as a solution to reduce vulnerability risks and accentuation of post-traumatic disorders.

## Ethics statement

My university does not currently have a formal ethics committee for research in the social sciences and humanities.

## Declaration of consent

As part of this research, particular attention was paid to ensuring the informed consent of all participants, in accordance with established ethical standards in the humanities and social sciences. Prior to each interview, participants were clearly and thoroughly informed of the study’s objectives, its purpose, the institutional framework, and the practical details of their involvement.

The information provided included:

✓ the voluntary nature of participation;
✓ the right to withdraw at any time without justification or consequence;
✓ the procedures for data collection, processing, and storage;
✓ assurances regarding anonymity and confidentiality;
✓ the absence of any foreseeable risk or negative impact for participants.

Each participant was given time to ask questions, request clarification, and reflect freely before making a decision. Where consent was given, it was recorded either orally. This process was designed to foster a climate of trust while ensuring that every participation was based on a full and freely given understanding of the aims and implications of the research.

## Data Availability

According to Law No. 001–2021/AN on the protection of individuals with regard to the processing of personal data in Burkina Faso, the sharing of raw data on internally displaced persons is strictly prohibited, as it violates the guarantee of anonymity and the security of the respondents. Therefore, the raw data (Excel file) from the questionnaire will be made available upon request to tengueri86@gmail.com. Zenodo: Trauma data and resilience, https://doi.org/10.5281/zenodo.19002544, [ Tengueri, Y. (2026b). Trauma data and resilience. Zenodo. https://doi.org/10.5281/zenodo.19002544]. The project contains the following underlying data: Trauma data and resilience. https://doi.org/10.5281/zenodo.19002544, (Types of violence experienced by Internally Displaced Women)
•
Figure 1_2_ *Pearson Correlation_ Recourse facing violence.*
• Map 1_ *Area covered by the survey* Zenodo: Trauma data and resilience, https://doi.org/10.5281/zenodo.19002544, [ Tengueri, Y. (2026b). Trauma data and resilience. Zenodo. https://doi.org/10.5281/zenodo.19002544]. This project contains the following extended data:
• Supplementary (Quantitative and qualitative Sample, professional status of IDW, number of dependent children by marital status of IDW, Pearson correlations between year of displacement and types of violence, Pearson correlations among types of violence, Human losses before and during forced displacement, Type of relationship with victims). • Supplementary (qualitative and quantitative data processing). • Questionnaire (questionnaire of types of violence against internally displaced women in the commune of Dédougou and interview Guide: Types of violence experienced by Internally Displaced Women). Data are available under the terms of the Creative Commons Zero “No rights reserved” data waiver (CC0 1.0 Public domain dedication).
