# Supplementary material for: Trauma and “spiritual resilience” among Internally Displaced Women (IDW) victims of violence in the commune of Dédougou, Burkina Faso
Source: Open Res Afr. 2026 Jun 29;9:38. [Version 1] doi: 10.12688/openresafrica.16517.1 (PMC13655436; doi:10.12688/openresafrica.16517.1)
Supplement: Peer review history

# Reviewer report: Reviewer response for version 1

**DOI:** 10.21956/openresafrica.17705.r36365

**Published:** 2026-10-12

**Authors:** Ramzan Shahid

**Affiliations:** 1 University of Gujrat, Gujrat City, Pakistan; 2 Arnold A. Saltzman Institute of War and Peace Studies, Columbia University, New York, USA, New York, New York, USA

It is very good study and covers most crucial issue of the region suffered by terrorism and political violence. The manuscript addresses an important and sensitive issue concerning the psychological trauma and spiritual resilience of internally displaced women who have experienced violence, including sexual violence and torture. The study is commendable for drawing attention to the experiences, suffering, and coping mechanisms of a particularly vulnerable population. It has the potential to make a valuable contribution to the existing literature on gender-based violence, forced displacement, trauma, and spiritual resilience.

 However, a few areas require improvement before indexing. The major concern relates to the literature review, which relies considerably on older sources. The authors should incorporate more recent scholarly literature to reflect contemporary research and developments in the field. Although some references from 2022 and 2023 have been included, the overall review would benefit from a more substantial engagement with recent studies, particularly those addressing the psychological and social consequences of violence, sexual abuse, forced displacement, and the role of spirituality in trauma recovery.

Is the work clearly and accurately presented and does it cite the current literature?

Partly

If applicable, is the statistical analysis and its interpretation appropriate?

I cannot comment. A qualified statistician is required.

Are all the source data underlying the results available to ensure full reproducibility?

Yes

Is the study design appropriate and is the work technically sound?

Yes

Are the conclusions drawn adequately supported by the results?

Yes

Are sufficient details of methods and analysis provided to allow replication by others?

Yes

Reviewer Expertise:

My area of research include; 1. Terrorism and Counter Terrorism 2. Political Violence 3.Security Studies 4. Geo-Politics

I confirm that I have read this submission and believe that I have an appropriate level of expertise to confirm that it is of an acceptable scientific standard.

# Reviewer report: Reviewer response for version 1

**DOI:** 10.21956/openresafrica.17705.r35196

**Published:** 2026-07-13

**Authors:** OUEDRAOGO Payaïssédé Salfo

**Affiliations:** 1 Université Joseph Ki-Zerbo, Ouagadougou, Burkina Faso

**Three Priority Points to Improve the Scientific Quality of the Article**

**1. Literature review overly international, insufficiently contextualised**

 A substantial portion of the introduction focuses on Nigeria, the DRC, Haiti and Iraq. While these cases are valuable for situating the study within a broader global context, the transition to the specific setting of Burkina Faso (Dédougou) requires clarification.

**Suggested improvement**: Add a bridging paragraph at the end of the introduction that synthesises the key lessons from the international literature and explicitly connects them to the Sahelian context, providing a brief overview of the security situation in Dédougou before moving into the study's research aim.

**2. Statistical contradiction between Table 4 and the accompanying text**

 Table 4 clearly states "No significant correlation" for social violence (r = -0.0498; p = 0.1126), yet the following text asserts the opposite: "there is a significant negative correlation... This significance suggests that...". Since p = 0.1126 exceeds the conventional threshold of 0.05, the table is correct. This is a verifiable factual error that must be corrected as a priority, as it undermines the credibility of the entire quantitative component and has knock-on effects in the discussion (notably the comparison with the OCADES 2022 report).

**Suggested improvement:** Revise the text to align with the table, explicitly stating that no significant relationship was found between year of displacement and social violence, and remove the associated interpretive claims.

**3. Overinterpretation of statistically significant correlations**

 The correlations presented as key findings (physical violence r = 0.0738; psychological violence r = 0.072) are extremely weak in terms of effect size, despite being statistically significant. With a sample of 1,015 participants, almost any non-zero correlation becomes significant, this does not mean it is substantively meaningful. The text should:

 •⁠  ⁠Clearly distinguish between statistical significance and practical significance (effect size);

 •⁠  ⁠Qualify interpretive statements (e.g. "the more recent the displacement, the greater the violence") that imply a stpubrong relationship, whereas r ≈ 0.07 accounts for only a negligible proportion of the variance.

**4. Methodological inconsistency regarding ethical safeguards**

 The study addresses extremely sensitive topics (forced infanticide, cannibalism, executions) without an institutional ethics committee or a documented protocol for participants' psychological protection (e.g. the right to pause or withdraw from the interview, immediate referral to psychological support). This gap must either be addressed or, at minimum, explicitly acknowledged and discussed as a major limitation of the study.

Is the work clearly and accurately presented and does it cite the current literature?

Yes

If applicable, is the statistical analysis and its interpretation appropriate?

Yes

Are all the source data underlying the results available to ensure full reproducibility?

Yes

Is the study design appropriate and is the work technically sound?

Yes

Are the conclusions drawn adequately supported by the results?

Yes

Are sufficient details of methods and analysis provided to allow replication by others?

Yes

Reviewer Expertise:

Migration, population displacement, public policy.

I confirm that I have read this submission and believe that I have an appropriate level of expertise to confirm that it is of an acceptable scientific standard.
